# LncRNA FOXD2-AS1 Functions as a Competing Endogenous RNA to Regulate TERT Expression by Sponging miR-7-5p in Thyroid Cancer

**DOI:** 10.3389/fendo.2019.00207

**Published:** 2019-04-08

**Authors:** Xiaoli Liu, Qingfeng Fu, Shijie Li, Nan Liang, Fang Li, Changlin Li, Chengqiu Sui, Gianlorenzo Dionigi, Hui Sun

**Affiliations:** ^1^Division of Thyroid Surgery, Jilin Provincial Key Laboratory of Surgical Translational Medicine, China-Japan Union Hospital of Jilin University, Changchun, China; ^2^Division for Endocrine and Minimally Invasive Surgery, Department of Human Pathology in Adulthood and Childhood “G. Barresi”, University Hospital “G. Martino”, University of Messina, Messina, Italy

**Keywords:** FOXD2-AS1, TERT, cancer stem cells, recurrence, thyroid cancer

## Abstract

Long non-coding RNA FOXD2 Adjacent Opposite Strand RNA 1 (FOXD2-AS1) has been widely reported to be implicated in the progression and recurrence of several cancers. The clinical significance and functional role of FOXD2-AS1 in thyroid carcinoma remain unknown. FOXD2-AS1 expression was evaluated by analyzing thyroid cancer RNA sequencing dataset from The Cancer Genome Atlas (TCGA). *In vitro* and *in vivo assays* were performed to assess the biological roles of FOXD2-AS1 in thyroid cancer cells. Western blot, luciferase, immunoprecipitation (IP), and RNA immunoprecipitation (RIP) assays were used to identify the underlying miRNA and mRNA target mediating the biological roles of FOXD2-AS1 in thyroid cancer cells. FOXD2-AS1 was upregulated in thyroid carcinoma tissues and cells. High expression of FOXD2-AS1 significantly correlated with clinical stage, recurrence of thyroid carcinoma. Silencing FOXD2-AS1 inhibited cancer stem cell-like phenotypes and attenuates the anoikis resistance *in vitro*. Downregulating FOXD2-AS1 represses the tumorigenesis of thyroid carcinoma cells *in vivo*. FOXD2-AS1 acts as a competitive endogenous RNA (ceRNA) for miR-7-5p, up-regulating the expression of telomerase reverse transcriptase (TERT), which further promotes the cancer stem cells features and anoikis resistance in thyroid cancer cells. Our findings indicate that FOXD2-AS1 functions as an oncogenic regulator in the development of thyroid cancer, contributing to early recurrence of thyroid cancer.

## Introduction

Thyroid cancer is the most frequent endocrine malignancy with an increasing incidence (1, 2). Among histological subtypes, papillary thyroid carcinoma (PTC) accounts for 90% of cases (3). PTCs have a favorable 5-year survival rate (over 95%) (4), however, about 5–10% PTC patients present recurrence after therapy (4, 5).

Long non-coding RNAs (lncRNAs) are newly identified class of non-coding RNA, longer than 200 nucleotides (6, 7). lncRNAs have been demonstrated to be involved in multiple biological processes, including transcription or post-transcription, epigenetic modification and mRNA processing (6, 7). Recently, accumulating studies have reported that lncRNAs play important role in the development, progression and metastasis of various types of cancer (8–11). However, literatures regarding the effects of lncRNAs on PTC are relatively scanty. The lncRNA, FOXD2 Adjacent Opposite Strand RNA 1 (FOXD2-AS1), has been reported to function as an oncogenic lncRNA in several human cancer types. For example, FOXD2-AS1 was found to be overexpressed in bladder cancer tissues, which further promoted bladder cancer progression and recurrence through forming a positive feedback loop with Akt and E2F1 (12); in addition, Xu et al. have reported that overexpression of FOXD2-AS1 contributed to carcinogenesis of gastric cancer, and predicted poor prognosis in gastric cancer patients (13). Furthermore, Lu's study has shown that FOXD-AS1 expression was significantly associated with overall survival in thyroid cancer patients (14). However, to date, the functional role of FOXD2-AS1 in the progression of thyroid cancer is unclear.

LncRNAs can function as a competing endogenous RNAs (ceRNAs, or a molecular sponge) to modulate microRNAs (miRNAs) expression, and focus on the miRNA-mediated lncRNA/mRNA crosstalk (15). miRNAs are a class of endogenous, small non-coding RNAs containing about 22 nucleotides that are involved in regulation of downstream target genes expression at a posttranscriptional level via binding with specific sequences in the 3′ untranslated region (3′UTR) of downstream target genes (16). Aberrant expression of miRNAs has been demonstrated to be involved in the tumorigenesis and metastasis of cancers (17–21). Studies have shown that miR-7-5p is downregulated in PTC (22–24). The aim of the current report is to evaluate the FOXD2-AS1 expression in thyroid cancer.

In the current study, we found that FOXD2-AS1 was upregulated in thyroid cancer tissues, and high levels of FOXD2-AS1 were positively associated with poor recurrence-free survival in thyroid cancer patients via analyzing thyroid cancer RNA sequencing dataset from The Cancer Genome Atlas (TCGA). Loss of function experiments revealed that silencing FOXD2-AS1 repressed CSCs characteristics and anoikis resistance in thyroid cancer cells *in vitro*, and tumorigenesis *in vivo*. Furthermore, mechanistic investigations revealed that FOXD2-AS1 functioned as a ceRNA by sponging miR-7-5p, which further upregulated telomerase reverse transcriptase (TERT) expression in thyroid cancer cells, finally contributing to the early recurrence of thyroid cancer. Therefore, our findings offer new insights into the molecular function of FOXD2-AS1 in thyroid cancer, supporting the notion that FOXD2-AS1 may be used as an early recurrent factor in thyroid cancer patients.

## Materials and Methods

### Cell Lines and Cell Culture

Normal primary thyroid follicular epithelial cells (PTFE) and thyroid duct cell carcinoma cells TT was purchased from Procell (Procell Life Science and Technology Co., Ltd., Wuhan, China). Thyroid cancer cell lines, including PTC cell lines (B-CPAP and BHT101), and anaplastic thyroid cancer (ATC) cell lines (CAL-62 and 8305C) were obtained from Cell Bank of Shanghai Institute of Cell Biology, Chinese Academy of Sciences (Shanghai, China). PTFE were cultured in CM-H023 medium (Procell, China), and thyroid cancer cell lines were cultured in RPMI-1640 medium (Life Technologies, Carlsbad, CA, US) supplemented with penicillin G (100 U/ml), streptomycin (100 mg/ml) and 10% fetal bovine serum (FBS, Life Technologies). All cell lines were cultured at 37°C in a humidified atmosphere with 5% CO_2_.

### RNA Extraction, Reverse Transcription, and Real-Time PCR

RNA from tissues and cells was extracted (TRIzol, Life Technologies) according to the manufacturer's instructions. Messenger RNA (mRNA), lncRNA and miRNA were reverse transcribed from the total RNA using the Revert Aid First Strand cDNA Synthesis Kit (Thermo, USA) according to the manufacturer's protocol. Complementary DNA (cDNA) was amplified and quantified on ABI 7500HT system (Applied Biosystems, Foster City, CA, USA) using SYBR Green I (Applied Biosystems). The primers used in the reactions are listed in Table 1. Real-time PCR was performed according to a standard method, as described previously (25). Primers for miR-7-5p and miR-7-1-3p were synthesized and purified by RiboBio (Guangzhou, China). U6 or glyceraldehyde-3-phosphate dehydrogenase (GAPDH) was used as endogenous controls for miRNA or mRNA, respectively. Relative fold expressions were calculated with the comparative threshold cycle method according to a previous study (26).

**Table 1:** A list of primers used in the reactions for real-time PCR.

**Gene**	**Sequence (5^**′**^-3^**′**^)**
FOXD2-AS1-up	ACTGGCTTGAAGCGGAGTTTG
FOXD2-AS1-dn	TTAGAGAAATCTGCGGGCGTAG
GAPDH-up	TCCTCTGACTTCAACAGCGACAC
GAPDH-dn	CACCCTGTTGCTGTAGCCAAATTC
NANOG-up	TCCAACATCCTGAACCTCAGCTA
NANOG-dn	AGTCGGGTTCACCAGGCATC
SOX2-up	GTGAGCGCCCTGCAGTACAA
SOX2-dn	GCGAGTAGGACATGCTGTAGGTG
OCT4-up	TGAAGCTGGAGAAGGAGAAGCTG
OCT4-dn	GCAGATGGTCGTTTGGCTGA
BMI-1-up	TCGTTGTTCGATGCATTTCT
BMI-1-dn	CTTTCATTGTCTTTTCCGCC
ABCG2-up	ATGAACACACATGTGCAACCATC
ABCG2-dn	CACAGAAACACAACACTTGGCTGTA
ALDH1A1-up	ACAGTGGTTGTCAAACCAGCAGAG
ALDH1A1-dn	TGTAGGCCCATAACCAGGAACAATA
KLF4-up	CCCCGTGTGTTTACGGTAGT
KLF4-dn	GAGTTCCCATCTCAAGGCAC
TERT-up	TCACGGAGACCACGTTTCAAA
TERT-dn	TTCAAGTGCTGTCTGATTCCAAT

### Plasmid, miRNA Inhibitor, and Transfection

Human FOXD2-AS1 cDNA (Vigene Biosciences, Shandong, China) was cloned into the pcDNA3.1(+) plasmid. Knockdown of endogenous FOXD2-AS1 was performed by cloning two short hairpin RNA (shRNA) oligonucleotides into the GV493 vector (GenChem, Shanghai, China). The sequences of the two separate shRNA fragments and scramble are listed in Table 2. The miR-7-5p inhibitor and the negative control (RiboBio, China) were achieved. Transfection of siRNAs and plasmids was performed as previously described (27).

**Table 2:** A list of primers used in the reactions for clone PCR.

**Gene**	**Sequence (5^**′**^-3^**′**^)**
shFOXD2-AS1-1#-up	CCGGCAGCGATTATGCGGATCTAATCTCGAG ATTAGA TCCGCATAATCGCTGTTTTTG
shFOXD2-AS1-1#-dn	AATTCAAAAACAGCGATTATGCGGATCTAA TCTCGAG ATTAGATCCGCATAATCGCTG
shFOXD2-AS1-2#-up	CCGGGGGCAAAGTTCGAGAGTGAATCTCGAG ATTCA CTCTCGAACTTTGCCCTTTTTG
shFOXD2-AS1-2#-dn	AATTCAAAAAGGGCAAAGTTCGAGAGTGAATC TCG AGATTCACTCTCGAACTTTGCCC
sh-Scramble-up	CCGGTTCTCCGAACGTGTCACGTCTCGAGAC GTGAC ACGTTCGGAGAATTTTTG
sh-Scramble-dn	AATTCAAAAATTCTCCGAACGTGTCACGTC TCGAGA CGTGACACGTTCGGAGAA

### Western Blotting Analysis

Western blot was performed according to a standard method (28). Antibodies against cytochrome C, ABCG2, SOX2, NANOG, BMI-1, and LSD1 (Cell Signaling Technology, Cambridge, USA) and TERT (Invitrogen, California, USA) were obtained. The membranes were re-probed with an anti–α-tubulin antibody (Cell Signaling Technology) as the loading control.

### Anchorage-Independent Growth Assay

Five-hundred cells were suspended in 2 ml of complete medium containing 0.3% agar (Sigma, Burlington, USA). This experiment was performed according to standards (29) and three times independently for each cell line.

### Flow Cytometric Analysis

Flow cytometric analysis (keyGen BioTECH) was performed as standards (30). The cell's inner mitochondrial membrane potential (Δψm) was detected by flow cytometric analysis using MitoScreen JC-1 staining kit (keyGen BioTECH). Briefly, cells were dissociated with trypsin and resuspended at 1 × 10^6^ cells/ml in Assay Buffer, and then incubated at 37°C for 15 min with 10 μl/ml JC-1. Before analyzed by the flow cytometer, cells were washed twice by Assay Buffer. Flow cytometric data were analyzed using FlowJo v10 software (TreeStar Inc., USA).

### Caspase-9 or Caspase-3 Activity Assays

The activity of caspase-9 or caspase-3 was analyzed by spectrophotometry (Keygen, China) (31). Briefly, 5 × 10^6^ cells or 100 mg fresh tumor tissues were washed with cold phosphate-buffered saline (PBS) and resuspended in Lysis Buffer and incubated in ice for 30 min. Mixed the 50 μl cell suspension, 50 μl Reaction Buffer, and 5 μl Caspase-3/-9 substrate, and then incubated at 37°C for 4 h. The absorbance was measured at 405 nm, and bicinchoninic acid (BCA) protein quantitative analysis was used as the reference.

### Side Population Analysis

The cell suspensions were labeled (Hoechst 33342, Molecular probes – #H-3570) and dye for side population analysis. Cells were resuspended at 1 × pre-warmed (OptiMEM, Gibco, USA) containing 2% fetal bovine serum (FBS) (Gibco, USA) at a density of 10^6^/mL. Hoechst 33342 dye was added at a final concentration of 5 mg/mL in the presence or absence of verapamil (50 mmol/L; Sigma) and the cells were incubated at 37°C for 90 min with intermittent shaking. At the end of the incubation, the cells were washed with OptiMem containing 2% FBS and centrifuged down at 4°C, and resuspended in ice-cold OptiMem containing 2% FBS and 10 mmol/L HEPES. Propidium iodide (Sigma, USA) at a final concentration of 2 mg/mL was added to the cells to gate viable cells. The cells were filtered through a 40-lm cell strainer to obtain single cell suspension before sorting. Analysis and sorting were done (FACS AriaI, Becton Dickinson). The Hoechst 33342 dye was excited at 355 nm and its dual-wavelength emission at blue and red region was plotted to get the side population (SP) scatter.

### Spheroid Formation Assay

Cells (500 cells/well) were seeded into 6-well plates (Ultra Low Cluster, Corning) and cultured (32). After 10–12 days, the number of cell spheroids (tight, spherical, non-adherent masses >50 μm in diameter) were counted, and images of the spheroids were scored under an inverse microscope (spheroids formation efficiency = colonies/input cells × 100%).

### RNA Immunoprecipitation Assay

Cells were co-transfected with pIRESneo-FLAG/HA-Ago2 (#10822; Addgene Inc., USA), followed by HA-Ago2 immunoprecipitation using HA-antibody (33). Real-time PCR analysis of the immunoprecipitation (IP) material was used to test the association of the miR-7-5p with the RNA-induced silencing complex (RISC).

### RNA Pull-Down Assay

Details of the RNA pull-down experiment were obtained (34). The proteins in the pull-down products were then examined by western blot technique.

### Tumor Xenografts

To study the effect of FOXD2-AS1 on the tumorigenesis of thyroid cancer cells, the 6-week-old BALB/c-nu mice were randomly divided into four groups (*n* = 6 per group). Cells (5 × 10^6^, 1 × 10^6^, 5 × 10^5^, and 1 × 10^5^) were inoculated subcutaneously together with Matrigel (final concentration of 25%) into the inguinal folds of the nude mice, respectively. Tumor volume was determined using an external caliper and calculated using the equation (L × W2)/2. On day 38, tumors were excised, weighed and stored in liquid nitrogen tanks. All the animal experimental procedures were performed in accordance with the guidelines of the Institutional Animal Care and Use Committee. The protocol was approved by the Animal Ethics Committee of the China-Japan Union Hospital of Jilin University.

### Statistical Analysis

All values are presented as means ± standard deviation (SD). Significant differences were determined using GraphPad 5.0 software (USA). Student's *t*-test was used to determine statistical differences between the two groups. One-way ANOVA was used to determine statistical differences between multiple testing. The chi-square test was used to analyze the relationship between FOXD2-AS1 expression and clinicopathological characteristics. Survival curves were plotted using the Kaplan Meier method and compared by log-rank test. *P* < 0.05 was considered significant. Experiments were repeated three times.

## Results

### FOXD2-AS1 Is Up-Regulated in Thyroid Cancer Tissues

**Figure 1:**
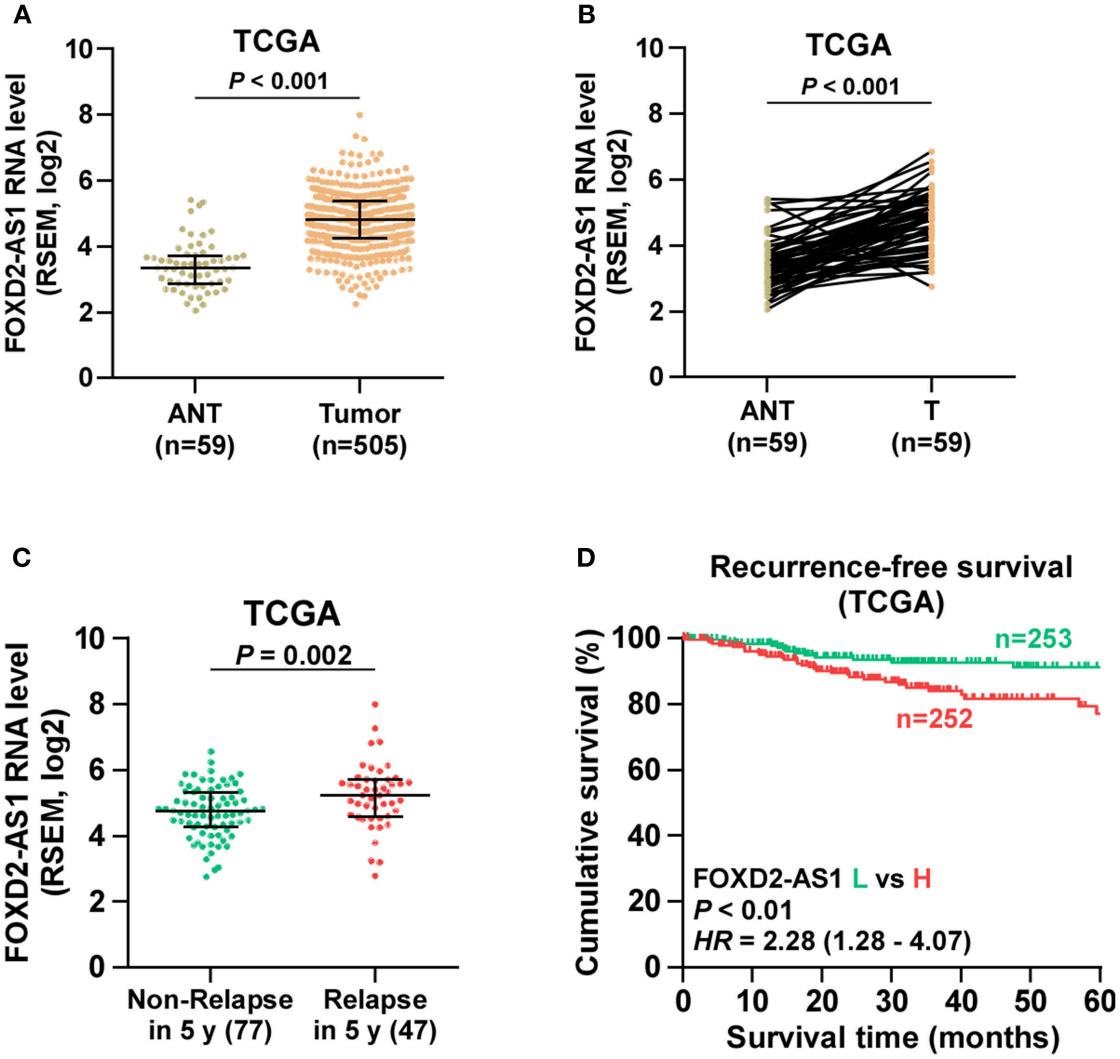
Overexpression of FOXD2-AS1 correlates with early recurrence in thyroid cancer patients. **(A)** FOXD2-AS1 expression in thyroid cancer tissues and the adjacent normal tissues (ANT) in the thyroid cancer dataset from TCGA. **(B)** FOXD2-AS1 expression in 59 paired thyroid cancer tissues and the matched adjacent normal tissues in the thyroid cancer dataset from TCGA. **(C)** FOXD2-AS1 expression in recurrent thyroid cancer tissues and non-recurrent thyroid cancer tissues in the thyroid cancer dataset from TCGA. **(D)** Kaplan-Meier analysis of FOXD2-AS1 expression in recurrence-free survival in the thyroid cancer dataset from TCGA. lncRNA FOXD2-AS1 expression levels in all thyroid cancer tissues were, respectively, normalized to that in the thyroid cancer tissue with the lowest level of lncRNA FOXD2-AS1 expression. Then, the median of lncRNA FOXD2-AS1 expression in thyroid cancer tissues was used as the cut off value to stratify high and low expression of lncRNA FOXD2-AS1.

Through analyzing RNA sequencing dataset of thyroid cancer from TCGA, we found that expression level of FOXD2-AS1 was increased in thyroid cancer tissues compared with the adjacent normal tissues (ANT) (Figure 1A). Furthermore, upregulation of FOXD2-AS1 in 59 paired thyroid cancer tissues was demonstrated compared with the matched ANT in the majority of thyroid cancer tissues (Figure 1B). Overexpression of FOXD2-AS1 was found to significantly correlate with age, T classification, N classification, clinical stage, and recurrence status in thyroid cancer patients via analyzing clinical dataset of thyroid cancer patients from TCGA (Table 3). Importantly, analysis result of TCGA showed that FOXD2-AS1 expression was significantly elevated in recurrent thyroid cancer tissues compared with those in non-recurrent thyroid cancer tissues (Figure 1C), and high expression of FOXD2-AS1 predicted poor recurrence-free survivals (Figure 1D). Therefore, these results suggest that overexpression of FOXD2-AS1 may be implicated in the early recurrence of thyroid cancer.

**Table 3:** The relationship between FOXD2-AS1 expression level and clinical pathological characteristics in 505 patients with thyroid carcinoma (from TCGA).

**Parameters**	**Number of cases**	**FOXD2-AS1 expression**		***P*-values**
		**Low**	**High**	
**HISTOLOGIC**				
PTC	495	248	247	0.995
ATC	10	5	5	
**GENDER**				
Male	136	64	72	0.407
Female	369	189	180	
**AGE**				
>55	338	180	158	0.044^*^
≤55	167	73	94	
**T CLASSIFICATION**				
T1-T2	309	173	136	0.001^*^
T3-T4	194	80	114	
**N CLASSIFICATION**				
N0	230	132	98	<0.001^*^
N1	225	88	137	
**M CLASSIFICATION**				
M0	282	135	147	0.18
M1	9	2	7	
**STAGE**				
I	224	116	108	0.004^*^
II-IV	64	20	44	
**RECURRENCE STATUS**				
No (>5 years)	79	46	33	0.003^*^
Yes (≤ 5 years)	46	14	32	

* *PTC, papillary thyroid carcinoma; ATC, anaplastic thyroid carcinoma; NA, Not available*.

### Silencing FOXD2-AS1 Attenuates CSCs Characteristics *in vitro* and *in vivo*

**Figure 2:**
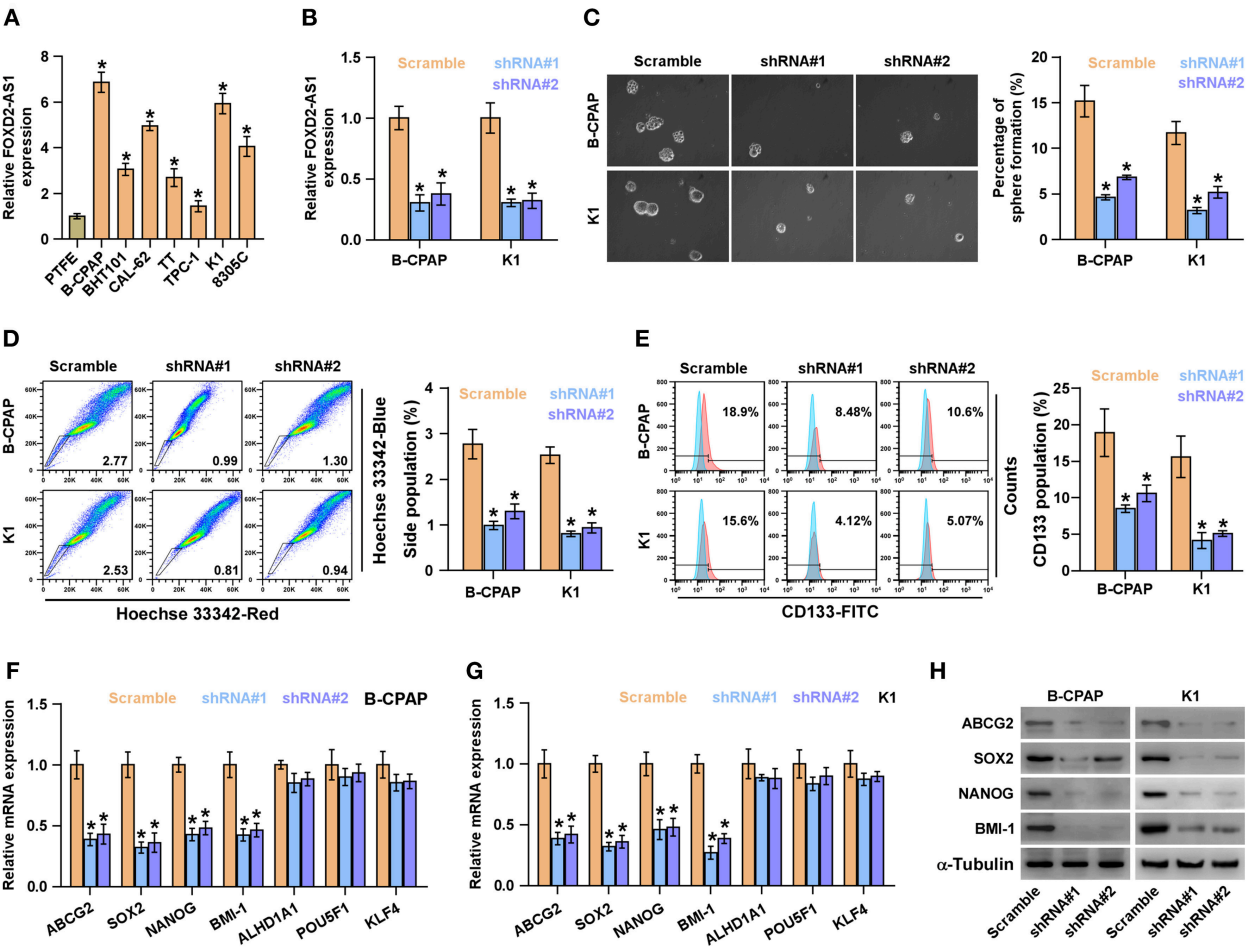
Silencing FOXD2-AS1 inhibits cancer stem cell characteristics in thyroid cancer cells. **(A)** Real-time PCR analysis of FOXD2-AS1 expression in normal thyroid follicular epithelial cells PTFE and seven thyroid cancer cells, including four PTC cell lines, B-CPAP, BHT101, KTC-1 and K1, and two ATC cell lines, CAL-62, and 8305C, and one thyroid duct cell carcinoma cells, TT. GAPDH was used as endogenous controls. *P < 0.05. **(B)** FOXD2-AS1 expression in the scramble, FOXD2-AS1 shRNA#1 and FOXD2-AS1 shRNA#2 thyroid cancer cells using real-time PCR. Transcript levels were normalized by GAPDH expression. *P < 0.05. **(C)** Representative images of spheroids formed by the scramble, FOXD2-AS1 shRNA#1 and FOXD2-AS1 shRNA#2 thyroid cancer cells at 200-fold magnification were counted. Histograms showed the mean number of spheroids formed. *P < 0.05. **(D)** The effect of silencing FOXD2-AS1 on side population in the indicated thyroid cancer cells by Hoechst 33342 dye exclusion assay. Histograms showed the fraction of side population in thyroid cancer cells.*P < 0.05**. (E)** The effect of silencing FOXD2-AS1 on the CD133^+^ population in the indicated thyroid cancer cells by flow cytometric analysis. Histograms showed the CD133^+^ percentage of thyroid cancer cells. *P < 0.05. **(F,G)** The effect of silencing FOXD2-AS1 on multiple stemness markers, including ABCG2, SOX2, NANOG, BMI-1, ALHD1A1, POU5F1, and KLF4, by real-time PCR analysis in the indicated thyroid cancer cells. Transcript levels were normalized by GAPDH expression. *P < 0.05. **(H)** The effect of silencing FOXD2-AS1 on ABCG2, SOX2, NANOG, and BMI-1 by Western blot in the indicated thyroid cancer cells. α-Tubulin was detected as a loading control.

Existence of CSCs has been reported to contribute to the recurrence of thyroid cancer (35). Therefore, the effects of FOXD2-AS1 on the CSCs phenotypes of thyroid cancer cells were further investigated. The expression levels of FOXD2-AS1 in normal thyroid follicular epithelial cells PTFE and seven thyroid cancer cells were first examined, the results showed that FOXD2-AS1 level was differentially upregulated in thyroid cancer cells compared with that in PTFE cells (Figure 2A). As B-CPAP and K1 cells expressed the highest levels of FOXD2-AS1, thus we further knocked down FOXD2-AS1 expression in B-CPAP and K1 cells by retrovirus infection (Figure 2B). Spheroids formation assay was performed and the results showed that silencing FOXD2-AS1 inhibited spheroids formation ability in thyroid cancer cells (Figure 2C). Moreover, downregulating FOXD2-AS1 decreased the fraction of side population (SP) cells and CD133^+^ population of thyroid cancer cells by flow cytometry (Figures 2D,E). RT-PCR analysis showed that silencing FOXD2-AS1 repressed the expression of ABCG2, SOX2, NANOG, and BMI-1, but not of ALHD1A1, POU5F1, and KLF4 expression (Figures 2F,G), which was further validated by western blot (Figure 2H).

**Figure 3:**
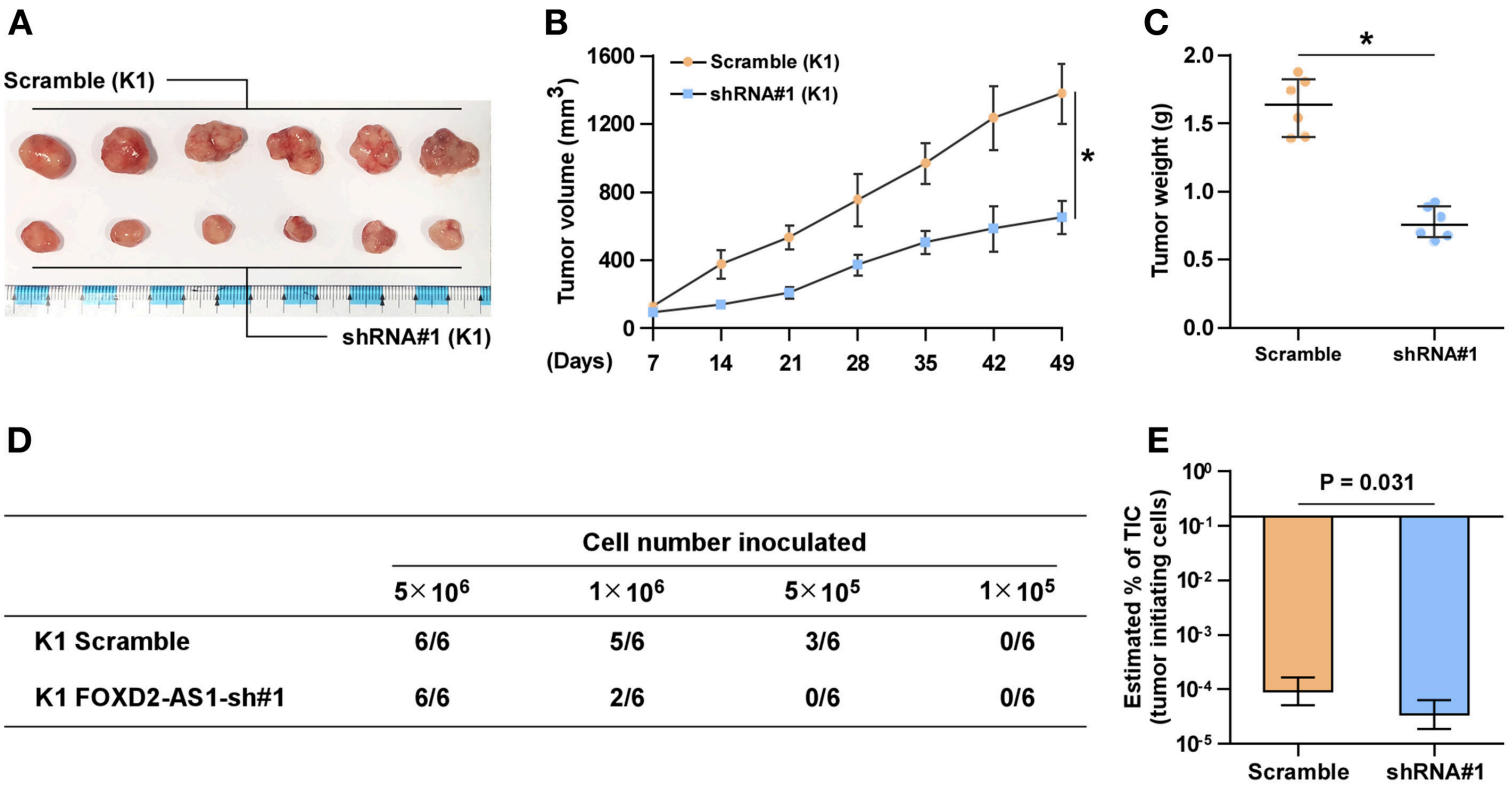
Downregulation of FOXD2-AS1 inhibits the CSCs characteristics *in vivo*. **(A)** The tumors formed by the scramble and FOXD2-AS1 shRNA#1 K1 cell were shown (*n* = 6, each group). **(B)** The effect of silencing FOXD2-AS1 on the tumor volumes in the indicated mice groups from the fifth day at 7 days interval after inoculation of 5 × 10^6^ cells. Data presented are the mean ± s.d. **(C)** The effect of silencing FOXD2-AS1 on the tumor weights in the indicated mice groups after inoculation of 5 × 10^6^ cells. *P < 0.05. **(D)** The formation number of tumors generated by different amounts of K1 cells in the indicated mice groups. **(E)** The estimated percentage of tumor-initiating cells required to generate tumors in the indicated mice groups.

The effect of FOXD2-AS1 on the tumorigenesis of thyroid cancer cells was further investigated *in vivo*. The results of animal experiments revealed that silencing FOXD2-AS1 reduced the volume and weight of tumors compared with those in the scramble group after implantation of 5 × 10^6^ cells (Figures 3A–C). Of note, after inoculation of 5 × 10^5^ scramble cells, the tumors were only detected in the scramble group compared with those in FOXD2-AS1-silencing group (Figure 3D). Furthermore, silencing FOXD2-AS1 increased the number of tumor initiating cells (TIC) required to develop tumor in mice compared with that in the scramble mice group (Figure 3E). Taken together, these results demonstrate that silencing FOXD2-AS1 inhibits CSCs characteristics *in vitro* and *in vivo*.

### Silencing FOXD2-AS1 Attenuates Survival and Anoikis Resistance in Thyroid Cancer Cells

**Figure 4:**
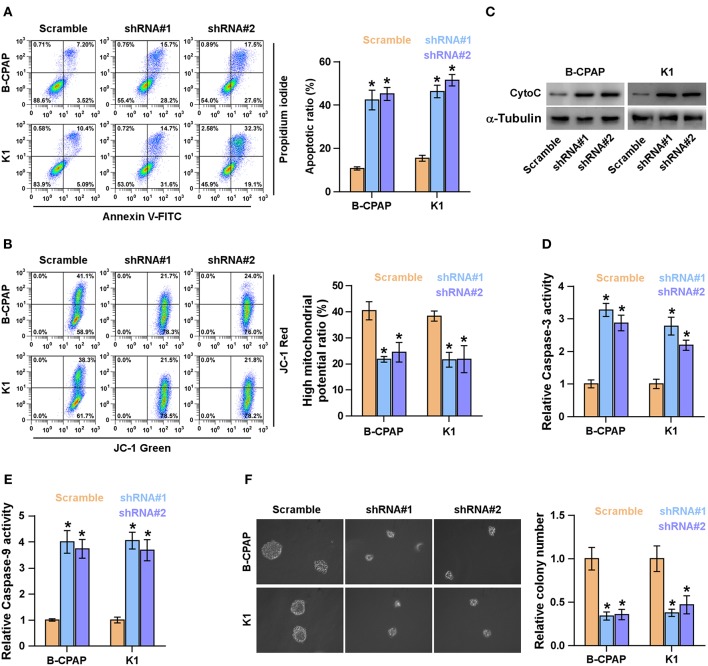
Silencing FOXD2-AS1 attenuates anoikis resistance in thyroid cancer cells. **(A)** The effect of silencing FOXD2-AS1 on the apoptotic ratio in the indicated thyroid cancer cells by Annexin V-FITC/PI staining. *P < 0.05. **(B)** The effect of silencing FOXD2-AS1 on mitochondrial potential in the indicated thyroid cancer cells by JC-1 staining. *P < 0.05. **(C)** Western blotting analysis of cytochrome C (CytoC) expression in the indicated thyroid cancer cells. α-Tubulin served as the loading control. **(D,E)** Analysis of the activities of caspase-3 **(D)** and caspase-9 **(E)** in the indicated thyroid cancer cells. *P < 0.05. **(F)** The effect of silencing FOXD2-AS1 on survival ability in the indicated thyroid cancer cells by anchorage-independent growth assay. *P < 0.05.

Anoikis resistance ability has been identified to be a major hallmark of TICs (36, 37). Therefore, the effects of FOXD2-AS1 on anoikis resistance of thyroid cancer cells were further examined. As shown in Figure 4A, silencing FOXD2-AS1 increased the apoptosis rate of thyroid cancer cells. In addition, silencing FOXD2-AS1 reduced the mitochondrial potential of thyroid cancer cells via mitochondrial membrane potential assay (Figure 4B). The effect of FOXD2-AS1 on the expression of cytochrome C (CytoC) and the caspase-3 and -9 activity were further examined, and we found that silencing FOXD2-AS1 enhanced the expression of CytoC and the activity of caspase-3 or -9 in thyroid cancer cells (Figures 4C–E). Anchorage-independent growth assay showed that silencing FOXD2-AS1 repressed the survival ability of thyroid cancer cells (Figure 4F). Collectively, these results indicate that silencing FOXD2-AS1 reduces anoikis resistance and survival in thyroid cancer cells.

### Silencing FOXD2-AS1 Down-Regulates TERT at the Post-transcriptional Level

**Figure 5:**
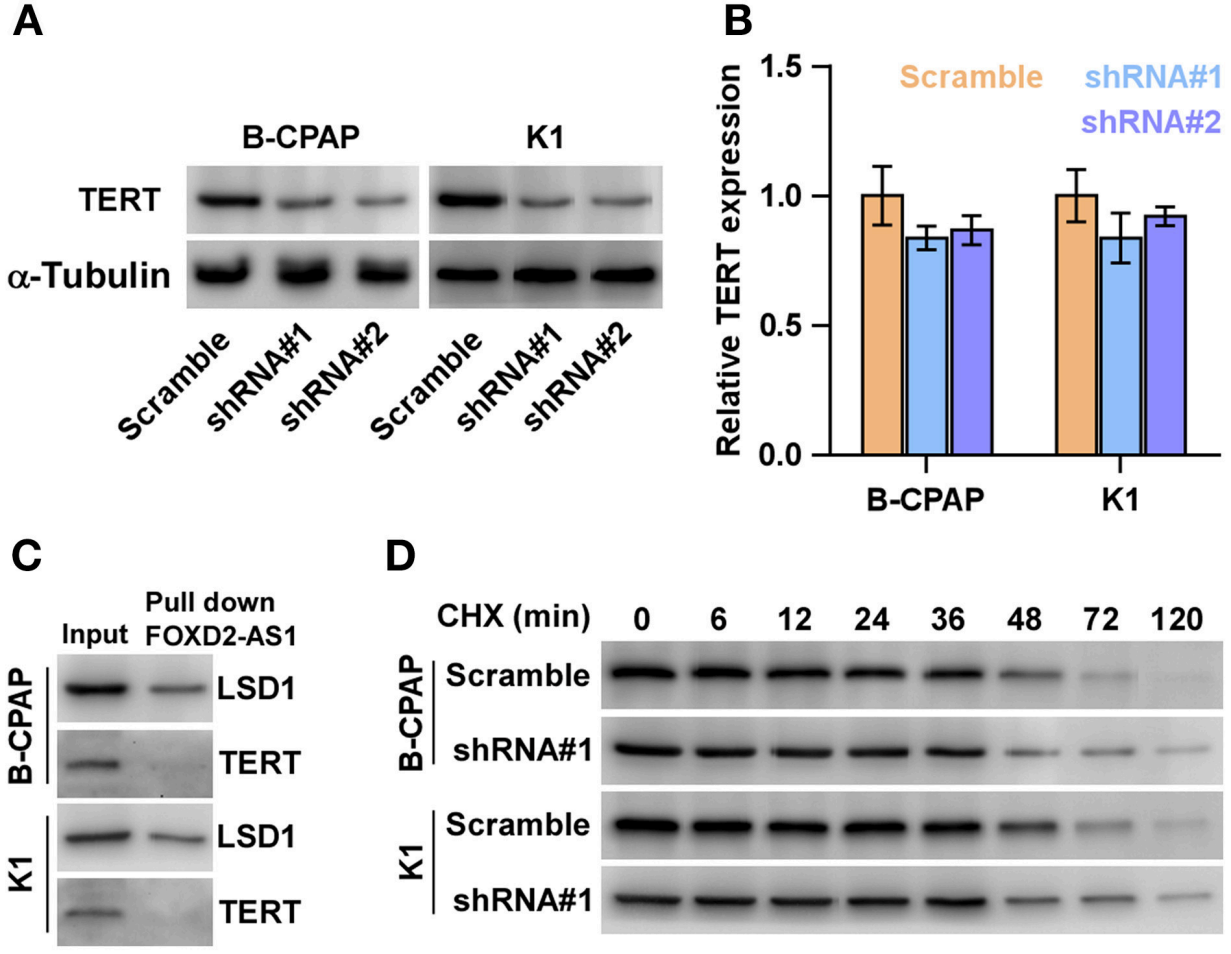
Silencing FOXD2-AS1 downregulates TERT. **(A)** Western blot analysis of TERT expression in the indicated thyroid cancer cells. α-Tubulin was detected as a loading control. **(B)** Real-time PCR analysis of TERT expression in the indicated thyroid cancer cells thyroid cancer cells. Transcript levels were normalized by GADPH expression. *P < 0.05. **(C)** Biotinylated FOXD2-AS1 was incubated with total extracts of thyroid cancer cells, targeted with streptavidin beads and the LSD1 or TERT proteins were analyzed by Western blot. LSD1 binding to FOXD2-AS1 was served as a positive control. **(D)** Half-life analysis of the TERT protein. Cells were treated with 20 μM cycloheximide (CHX) in the indicated times and then analyzed by Western blotting.

Several studies have demonstrated that overexpression of TERT is not only involved the development and aggressive behaviors of thyroid cancer cells (38), but also predicts early recurrence in thyroid cancer patients (39). Furthermore, promoter mutation of TERT has been identified to be significantly correlated with aggressiveness and recurrence in thyroid cancer in our previous studies (39–41). Therefore, we posited that FOXD2-AS1 promotes the recurrence of thyroid cancer via regulating TERT expression. As expected, TERT expression was repressed by downregulation of FOXD2-AS1 in thyroid cancer cells (Figure 5A). Strikingly, the mRNA level of TERT was not affected by FOXD2-AS1 (Figure 5B), suggesting that FOXD2-AS1 post-transcriptionally regulated TERT expression. A major role of lncRNAs in modulating downstream factors is as RNA-binding proteins (34, 41) such as LSD1 (42). The interactions between FOXD2-AS1 and LSD1 was confirmed by RNA pull-down assay, but FOXD2-AS1 had no interaction with TERT (Figure 5C). The half-life period of TERT was not affected by FOXD2-AS1 in thyroid cancer cells (Figure 5D). Thus, these findings indicate that some unknown post-transcriptional regulatory mechanism may be implicated in the regulatory role of FOXD2-AS1 in TERT in thyroid cancer cells.

### FOXD2-AS1 Functions as a ceRNA to Sponge miR-7-5p

**Figure 6:**
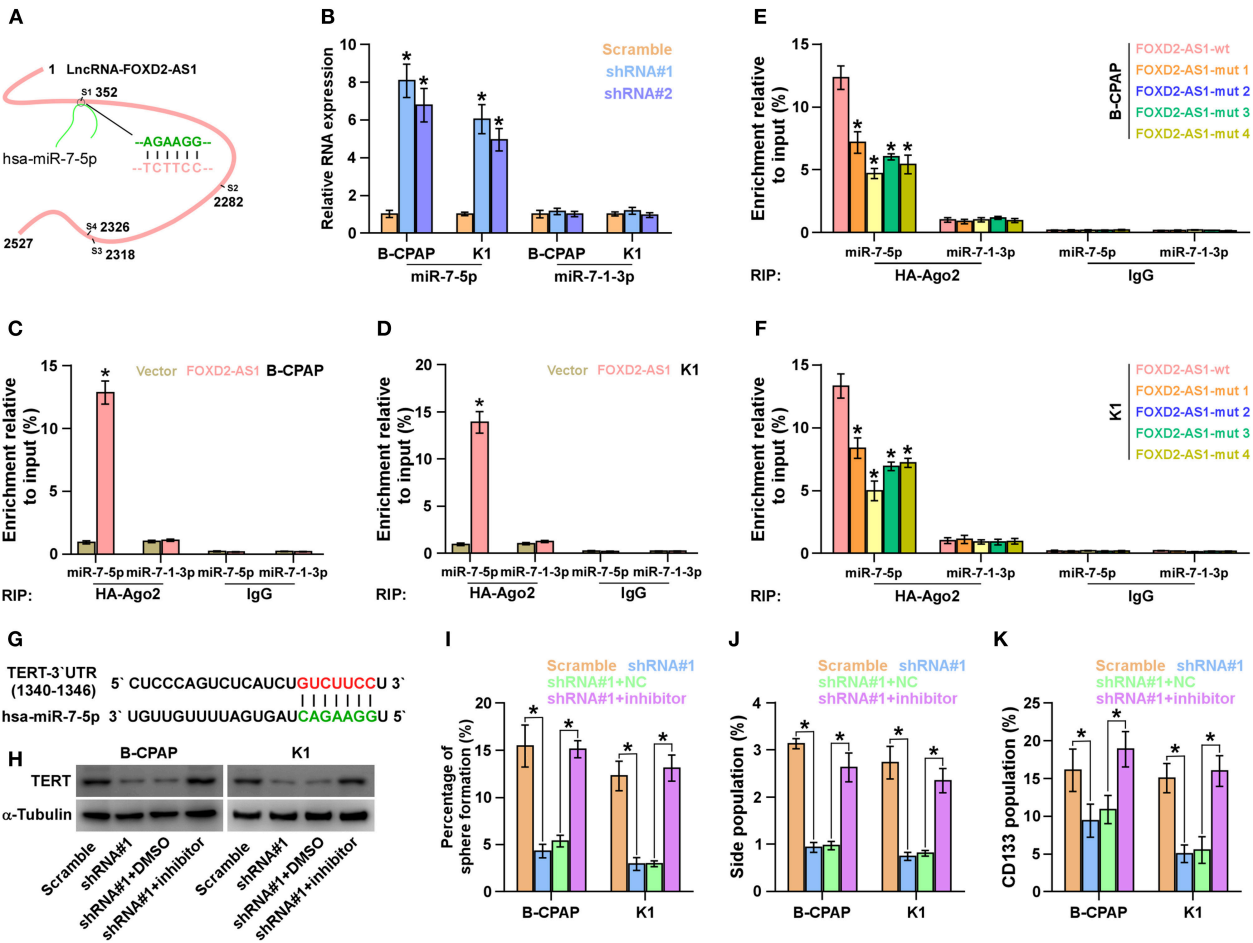
FOXD2-AS1 functions as a ceRNA to sponge miR-7-5p to regulate TERT expression. **(A)** Predicted recognition sites of miR-7-5p on FOXD2-AS1. **(B)** Real-time PCR analysis of miR-7-5p and miR-7-1-3p expression in the indicated thyroid cancer cells. Transcript levels were normalized by *U6* expression. Error bars represent the mean ± s.d. of three independent experiments. *P < 0.05. **(C,D)** The interaction between wild-type FOXD2-AS1 and miR-7-5p or miR-7-1-3p in thyroid cancer cells was investigated by RNA immunoprecipitation assay. Real-time PCR was performed to examine the expression of miR-7-5p and miR-7-1-3p. *P < 0.05. **(E,F)** The interaction between the wild-type or mutant FOXD2-AS1 and miR-7-5p or miR-7-1-3p in thyroid cancer cells was investigated by RNA immunoprecipitation assay. Real-time PCR was performed to examine the expression of miR-7-5p and miR-7-1-3p. *P < 0.05. **(G)** The predicted miR-7-5p target sequence in 3'UTRs of TERT. **(H)** Western blotting of TERT expression in the indicated cells. α-Tubulin served as the loading control. **(I)** Inhibition of miR-7-5p reversed the inhibitory effects of silencing FOXD2-AS1 on sphere formation ability of thyroid cancer cells. *P < 0.05. **(J)** Inhibition of miR-7-5p reversed the inhibitory effects of silencing FOXD2-AS1 on the fraction of side population in thyroid cancer cells. *P < 0.05. **(K)** Inhibition of miR-7-5p reversed the inhibitory effects of silencing FOXD2-AS1 on the fraction of CD133^+^ in thyroid cancer cells. *P < 0.05.

Using the publicly available algorithm TargetScan, we found that miR-7-5p that has been reported to act as a tumor-suppressive miRNA in thyroid cancer (42, 43) had 4 miRNA recognition sequences on FOXD2-AS1, suggesting that miR-7-5p was a potential target of FOXD2-AS1 (Figure 6A). RT-PCR analysis showed that silencing FOXD2-AS1 increased miR-7-5p, but not miR-7-1-3p, expression in thyroid cancer cells (Figure 6B). The interaction of FOXD2-AS1 with miR-7-5p was further analyzed by RNA immunoprecipitation (RIP) experiment, and the results showed that FOXD2-AS1 was enriched in Ago2-contaning miR-7-5p immunoprecipitate compared with control immunoglobulin G (IgG) or miR-7-1-3p immunoprecipitates (Figures 6C,D). Moreover, RIP results indicated that mutation of any four recognition site of miR-7-5p on FOXD2-AS1 differentially reduced the binding of miR-7-5p with FOXD2-AS1 compared with wild-type FOXD2-AS1 in thyroid cancer cells (Figures 6E,F). Therefore, these findings indicate that FOXD2-AS1 may act as a ceRNA to regulate miR-7-5p expression in thyroid cancer cells.

### TERT Is a Target of miR-7-5p

Interestingly, TERT was found to be a potential target of miR-7-5p (Figure 6G). Silencing FOXD2-AS1 dramatically reduced the TERT expression (Figure 6H). Importantly, inhibition of miR-7-5p increased the TERT expression in FOXD2-AS1-silenced thyroid cancer cells (Figure 6H). Thus, our results demonstrate that FOXD2-AS1 functions as a ceRNA sponge to disrupt the inhibitory effect of miR-7-5p on TERT, finally upregulating TERT expression in thyroid cancer cells.

### The Effects of FOXD2-AS1 on CSCs Features of Thyroid Cancer Cells Depend on miR-7-5p

We further investigated whether FOXD2-AS1 has an effect on CSCs phenotype of thyroid cancer cells via sponging miR-7-5p, and found that inhibition of miR-7-5p enhanced the sphere formation ability, SP fraction and CD133^+^ population repressed by downregulation of FOXD2-AS1 (Figures 6I–K). Therefore, our results indicate that FOXD2-AS1 promotes CSCs features by competitively binding miR-7-5p in thyroid cancer cells.

### Correlation of FOXD2-AS1 With miR-7-5p and TERT

**Figure 7:**
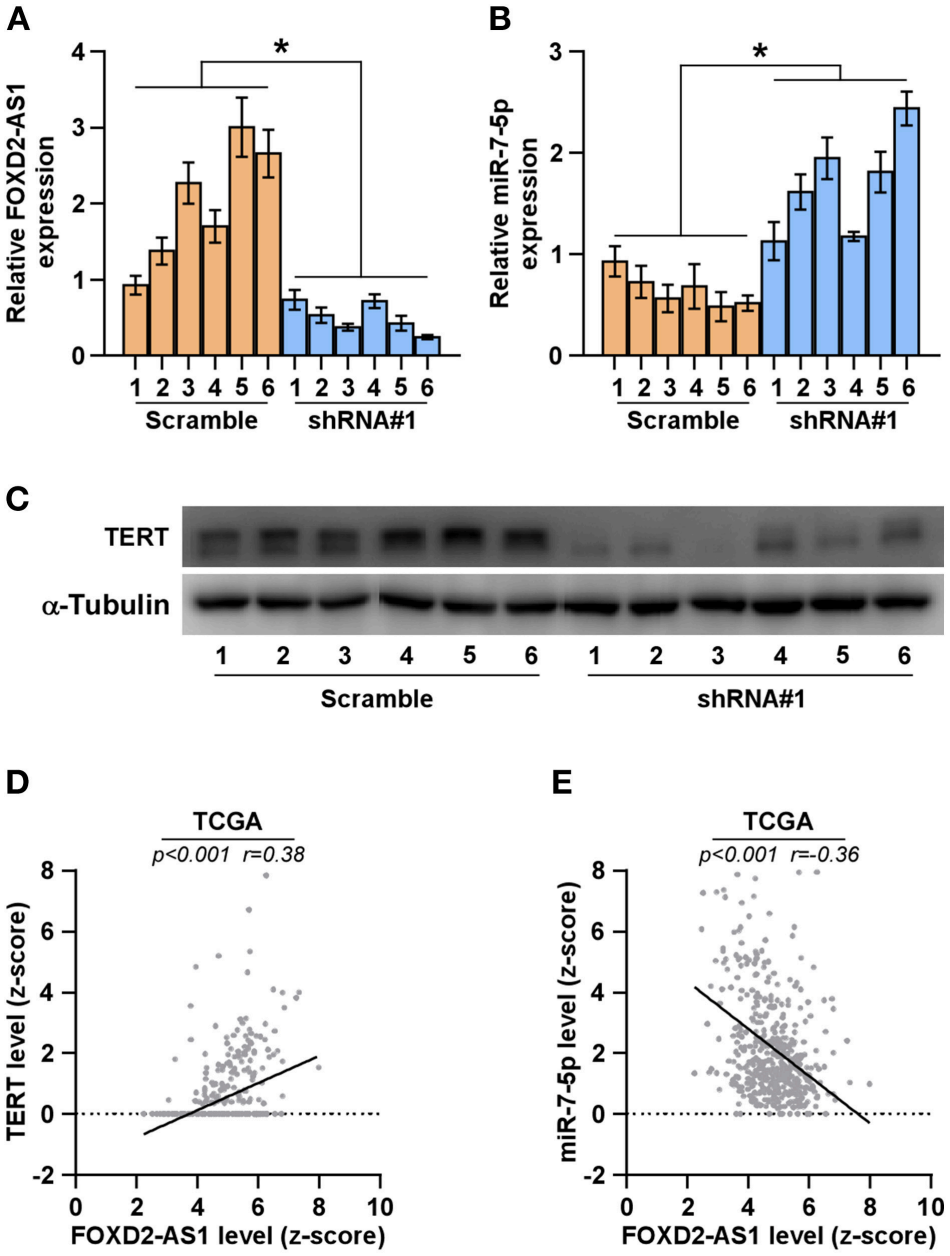
Correlation of FOXD2-AS1 with miR-7-5p and TERT **(A,B)** FOXD2-AS1 and miR-7-5p expression levels in the tumors from the scramble and FOXD2-AS1 shRNA#1 group of mice were determined by real-time PCR analysis. FOXD2-AS1 and miR-7-5p expression levels were normalized to that FOXD2-AS1 expression of sample one. *P < 0.05. **(C)** TERT expression levels in the tumors from the scramble and FOXD2-AS1 shRNA#1 groups of mice were examined by Western blot analysis. α-tubulin was used as the loading controls. **(D,E)** Correlation between FOXD2-AS1, miR-7-5p and TERT in clinical thyroid cancer samples from TCGA.

The clinical association of FOXD2-AS1 with miR-7-5p and TERT were further investigated in the tumors tissues of mice formed by 5 × 10^6^ FOXD2-AS1-silencing cells and the scramble cells. RT-PCR was performed to measure the expression of FOXD2-AS1 and miR-7-5p in the tumor tissues, and the results showed that FOXD2-AS1 expression levels were decreased in the tumor tissues from FOXD2-AS1-silencing mice group compared with those from the scramble mice group; conversely, miR-7-5p expression levels were elevated (Figures 7A,B). Furthermore, TERT expression was reduced in the tumor tissues from FOXD2-AS1-silencing mice group compared with those from the scramble mice group (Figure 7C). In addition, we further analyzed the correlation between FOXD2-AS1, miR-7-5p, and TERT expression in clinical samples from TCGA, and found that FOXD2-AS1 expression levels significantly and positively correlated with TERT expression (Figure 7D), and was negatively associated with miR-7-5p in thyroid cancer tissues (Figure 7E). Collectively, our results demonstrate that TERT expression is negatively associated with miR-7-5p expression and positively correlated with FOXD2-AS1 expression.

## Discussion

The clinical significance and biological function of FOXD2-AS1 in thyroid cancer are under-reported unknown. In the current study, we found that FOXD2-AS1 was upregulated in thyroid cancer tissues, and high expression of FOXD2-AS1 predicted recurrence in thyroid cancer patients. Silencing FOXD2-AS1 abrogated the CSCs-like phenotypes of thyroid cancer cells *in vitro* and the tumorigenesis of thyroid cancer cells *in vivo*. Therefore, our findings suggest the oncogenic role of FOXD2-AS1 in thyroid cancer.

The underlying mechanisms by which lncRNAs promote the tumorigenesis and metastasis of cancer involves transcription or post-transcription, epigenetic modification and mRNA processing (6, 7). It is now increasingly acknowledged that lncRNAs regulate development and progression via sponging an array of downstream miRNAs. Indeed, FOXD2-AS1 has been extensively reported to sponge a mass of miRNAs in a variety of cancers. In bladder cancer, FOXD2-AS1 was found to be overexpressed in bladder cancer tissues, which further promoted bladder cancer progression and recurrence through forming a positive feedback loop with Akt and E2F1 (12). In gastric cancer, FOXD2-AS1 acted as a molecular sponge of miR-136 to promote cancer overexpression of FOXD2-AS1 contributed to carcinogenesis of gastric cancer and predicted poor prognosis in gastric cancer patients (13).

Our results found that silencing FOXD2-AS1 reduced the protein, but not the mRNA, expression levels of TERT in thyroid cancer cells, suggesting that FOXD2-AS1 regulates TERT expression at post-transcriptional level. Telomerase reverse transcriptase (TERT, or hTERT) is a catalytic subunit of the telomerase enzyme, comprising the most important unit of the telomerase complex (44), and its aberrant expression has been widely reported in a variety of cancer types (45, 46). Of note, studies have reported that overexpression of TERT not only promotes the development and aggressive behaviors of thyroid cancer cells (38), but also predicts early recurrence in thyroid cancer patients (39). Our previous studies have widely demonstrated that the promoter mutation of TERT was significantly correlated with aggressiveness and recurrence in thyroid cancer (39–41). Bioinformatics analysis revealed the four recognition sequences of miR-7-5p on FOXD2-AS1. RT-PCR showed that silencing FOXD2-AS1 enhanced the expression of miR-7-5p in thyroid cancer cells. Downregulating miR-7-5p abolished the inhibitory effects of FOXD2-AS1 down-expression on the CSC phenotypes of thyroid cancer cells. In addition, FOXD2-AS1 acted as an endogenous sponge or decoy for miR-7-5p, which further relieved the inhibitory effects of miR-7-5p on TERT, leading to the recurrence of thyroid cancer patients. Therefore, our results unravel a novel mechanism underlying the oncogenic role of FOXD2-AS1 in thyroid cancer.

It has been extensively reported that lncRNAs can serve as a prognostic marker in thyroid cancer. Li et al. have found four-lncRNA signature complex as an independent prognostic predictor in PTC patients by genome-wide analysis of lncRNA expression profiles in a large cohort of PTC patients from TCGA (47). Consistently, a study from Luo et al. have revealed that lncRNAs, including AC079630.2, CRNDE, and CTD-2171N6.1, were closely related to the progression and survival of PTC, suggesting that this lncRNA may serve as a potential biomarker to predict the survival of PTC patient (48).

Lu's study has shown that FOXD-AS1 expression was significantly associated with overall survival in thyroid cancer patients (14). In this study, we identified that lncRNA FOXD2-AS1 expression was elevated in thyroid cancer tissues, particularly in recurrent thyroid cancer tissues. Importantly, Kaplan-Meier survival analysis showed that thyroid cancer patients with high expression of FOXD2-AS1 displayed early recurrence compared with those with low expression of FOXD2-AS1.

In summary, our study demonstrates that FOXD2-AS1 functions as a competing endogenous RNA to upregulate TERT expression by sponging miR-7-5p in thyroid cancer. Therefore, our results provide new insights into the mechanism that clarifies the function of FOXD2-AS1 in thyroid cancer, supporting the idea that FOXD2-AS1 can serve as a novel recurrent marker or a potential target in thyroid cancer.

## Ethics Statement

All the animal experimental procedures were performed in accordance with the guidelines of the Institutional Animal Care and Use Committee. The protocol was approved by the Animal Ethics Committee of the China-Japan Union Hospital of Jilin University.

## Author Contributions

XL and HS designed the study. XL, QF, SL, NL, FL, CL, and CS performed the experiments and acquired the data. XL, QF, SL, and NL analyzed and interpreted the results. XL, GD, and HS wrote the manuscript.

### Conflict of Interest Statement

The authors declare that the research was conducted in the absence of any commercial or financial relationships that could be construed as a potential conflict of interest.

## References

[1] Albores-Saavedra J Henson DE Glazer E Schwartz AM. Changing patterns in the incidence and survival of thyroid cancer with follicular phenotype–papillary, follicular, and anaplastic: a morphological and epidemiological study. Endocr Pathol. (2007) 18:1–7. 10.1007/s12022-007-0002-z 17652794

[2] Blomberg M Feldt-Rasmussen U Andersen KK Kjaer SK. Thyroid cancer in Denmark 1943-2008, before and after iodine supplementation. Int J Cancer. (2012) 131:2360–6. 10.1002/ijc.27497 22337133

[3] Mao Y Xing M. Recent incidences and differential trends of thyroid cancer in the USA. Endocr Relat Cancer. (2016) 23:313–22. 10.1530/ERC-15-0445 26917552 PMC4891202

[4] Hay ID Thompson GB Grant CS Bergstralh EJ Dvorak CE Gorman CA. Papillary thyroid carcinoma managed at the Mayo Clinic during six decades (1940-1999): temporal trends in initial therapy and long-term outcome in 2444 consecutively treated patients. World J Surg. (2002) 26:879–85. 10.1007/s00268-002-6612-1 12016468

[5] Mazzaferri EL Kloos RT. Clinical review 128: Current approaches to primary therapy for papillary and follicular thyroid cancer. J Clin Endocrinol Metab. (2001) 86:1447–63. 10.1210/jcem.86.4.7407 11297567

[6] Ponting CP Oliver PL Reik W. Evolution and functions of long noncoding RNAs. Cell. (2009) 136:629–41. 10.1016/j.cell.2009.02.006 19239885

[7] Salehi S Taheri MN Azarpira N Zare A Behzad-Behbahani A. State of the art technologies to explore long non-coding RNAs in cancer. J Cell Mol Med. (2017) 21:3120–3140. 10.1111/jcmm.13238 28631377 PMC5706582

[8] Lu CW Zhou DD Xie T Hao JL Pant OP Lu CB. HOXA11 antisense long noncoding RNA (HOXA11-AS): a promising lncRNA in human cancers. Cancer Med. (2018) 7:3792–9. 10.1002/cam4.1571 29992790 PMC6089141

[9] Gibb EA Brown CJ Lam WL. The functional role of long non-coding RNA in human carcinomas. Mol Cancer. (2011) 10:38. 10.1186/1476-4598-10-38 21489289 PMC3098824

[10] Peng WX Koirala P Mo YY. LncRNA-mediated regulation of cell signaling in cancer. Oncogene. (2017) 36:5661–7. 10.1038/onc.2017.184 28604750 PMC6450570

[11] Pan Z Mao W Bao Y Zhang M Su X Xu X. The long noncoding RNA CASC9 regulates migration and invasion in esophageal cancer. Cancer Med. (2016) 5:2442–7. 10.1002/cam4.770 27431358 PMC5055159

[12] Su F He W Chen C Liu M Liu H Xue F. The long non-coding RNA FOXD2-AS1 promotes bladder cancer progression and recurrence through a positive feedback loop with Akt and E2F1. Cell Death Dis. (2018) 9:233. 10.1038/s41419-018-0275-9 29445134 PMC5833400

[13] Xu TP Wang WY Ma P Shuai Y Zhao K Wang YF. Upregulation of the long noncoding RNA FOXD2-AS1 promotes carcinogenesis by epigenetically silencing EphB3 through EZH2 and LSD1, and predicts poor prognosis in gastric cancer. Oncogene. (2018) 37:5020–36. 10.1038/s41388-018-0308-y 29789713

[14] Lu W Xu Y Xu J Wang Z Ye G. Identification of differential expressed lncRNAs in human thyroid cancer by a genome-wide analyses. Cancer Med. (2018) 7:3935–44. 10.1002/cam4.1627 29923329 PMC6089163

[15] Li W Li N Shi K Chen Q. Systematic review and meta-analysis of the utility of long non-coding RNA GAS5 as a diagnostic and prognostic cancer biomarker. Oncotarget. (2017) 8:66414–25. 10.18632/oncotarget.19040 29029523 PMC5630423

[16] Bartel DP. MicroRNAs: target recognition and regulatory functions. Cell. (2009) 136:215–33. 10.1016/j.cell.2009.01.002 19167326 PMC3794896

[17] Guo W Ren D Chen X Tu X Huang S Wang M. HEF1 promotes epithelial mesenchymal transition and bone invasion in prostate cancer under the regulation of microRNA-145. J Cell Biochem. (2013) 114:1606–15. 10.1002/jcb.24502 23355420

[18] Ren D Wang M Guo W Huang S Wang Z Zhao X. Double-negative feedback loop between ZEB2 and miR-145 regulates epithelial-mesenchymal transition and stem cell properties in prostate cancer cells. Cell Tissue Res. (2014) 358:763–78. 10.1007/s00441-014-2001-y 25296715

[19] Longqiu Y Pengcheng L Xuejie F Peng Z. A miRNAs panel promotes the proliferation and invasion of colorectal cancer cells by targeting GABBR1. Cancer Med. (2016) 5:2022–31. 10.1002/cam4.760 27230463 PMC4884921

[20] Hu Y Wang H Chen E Xu Z Chen B Lu G. Candidate microRNAs as biomarkers of thyroid carcinoma: a systematic review, meta-analysis, and experimental validation. Cancer Med. (2016) 5:2602–14. 10.1002/cam4.811 27465286 PMC5055193

[21] Ren D Yang Q Dai Y Guo W Du H Song L. Oncogenic miR-210-3p promotes prostate cancer cell EMT and bone metastasis via NF-kappaB signaling pathway. Mol Cancer. (2017) 16:117. 10.1186/s12943-017-0688-6 28693582 PMC5504657

[22] Jahanbani I Al-Abdallah A Ali RH Al-Brahim N Mojiminiyi O. Discriminatory miRNAs for the management of papillary thyroid carcinoma and noninvasive follicular thyroid neoplasms with papillary-like nuclear features. Thyroid. (2018) 28:319–27. 10.1089/thy.2017.0127 29378472

[23] Saiselet M Gacquer D Spinette A Craciun L Decaussin-Petrucci M Andry G. New global analysis of the microRNA transcriptome of primary tumors and lymph node metastases of papillary thyroid cancer. BMC Genomics. (2015) 16:828. 10.1186/s12864-015-2082-3 26487287 PMC4618137

[24] Stokowy T Wojtaś B Fujarewicz K Jarza̧b B Eszlinger M Paschke R. miRNAs with the potential to distinguish follicular thyroid carcinomas from benign follicular thyroid tumors: results of a meta-analysis. Horm Metab Res. (2014) 46:171–80. 10.1055/s-0033-1363264 24446156

[25] Ren D Dai Y Yang Q Zhang X Guo W Ye L. Wnt5a induces and maintains prostate cancer cells dormancy in bone. J Exp Med. (2019) 216:428–49. 10.1084/jem.20180661 30593464 PMC6363426

[26] Dai Y Ren D Yang Q Cui Y Guo W Lai Y. The TGF-beta signalling negative regulator PICK1 represses prostate cancer metastasis to bone. Br J Cancer. (2017) 117:685–94. 10.1038/bjc.2017.212 28697177 PMC5572169

[27] Wu N Ren D Li S Ma W Hu S Jin Y. RCC2 over-expression in tumor cells alters apoptosis and drug sensitivity by regulating Rac1 activation. BMC Cancer. (2018) 18:67. 10.1186/s12885-017-3908-y 29321004 PMC5763756

[28] Ren D Wang M Guo W Zhao X Tu X Huang S. Wild-type p53 suppresses the epithelial-mesenchymal transition and stemness in PC-3 prostate cancer cells by modulating miR145. Int J Oncol. (2013) 42:1473–81. 10.3892/ijo.2013.1825 23404342

[29] Zhang X Ren D Guo L Wang L Wu S Lin C. Thymosin beta 10 is a key regulator of tumorigenesis and metastasis and a novel serum marker in breast cancer. Breast Cancer Res. (2017) 19:15. 10.1186/s13058-016-0785-2 28179017 PMC5299657

[30] Ren D Lin B Zhang X Peng Y Ye Z Ma Y. Maintenance of cancer stemness by miR-196b-5p contributes to chemoresistance of colorectal cancer cells via activating STAT3 signaling pathway. Oncotarget. (2017) 8:49807–23 10.18632/oncotarget.17971 28591704 PMC5564809

[31] Zhang X Ren D Wu X Lin X Ye L Lin C. miR-1266 contributes to pancreatic cancer progression and chemoresistance by the STAT3 and NF-kappaB signaling pathways. Mol Ther Nucleic Acids. (2018) 11:142–58. 10.1016/j.omtn.2018.01.004 29858050 PMC5842289

[32] Zhang X Zhang L Lin B Chai X Li R Liao Y. Phospholipid Phosphatase 4 promotes proliferation and tumorigenesis, and activates Ca2+-permeable Cationic Channel in lung carcinoma cells. Mol Cancer. (2017) 16:147. 10.1186/s12943-017-0717-5 28851360 PMC5576330

[33] Li X Liu F Lin B Luo H Liu M Wu J miR150 inhibits proliferation and tumorigenicity via retarding G1/S phase transition in nasopharyngeal carcinoma. Int J Oncol. (2017) 50:1097–1108. 10.3892/ijo.2017.3909 PMC5363880 28350089

[34] Xu TP Liu XX Xia R Yin L Kong R Chen WM. SP1-induced upregulation of the long noncoding RNA TINCR regulates cell proliferation and apoptosis by affecting KLF2 mRNA stability in gastric cancer. Oncogene. (2015) 34:5648–61. 10.1038/onc.2015.18 25728677

[35] Ke CC Liu RS Yang AH Liu CS Chi CW Tseng LM. CD133-expressing thyroid cancer cells are undifferentiated, radioresistant and survive radioiodide therapy. Eur J Nucl Med Mol Imaging. (2013) 40:61–71. 10.1007/s00259-012-2242-5 23081821 PMC3510415

[36] Haemmerle M Taylor ML Gutschner T Pradeep S Cho MS Sheng J. Platelets reduce anoikis and promote metastasis by activating YAP1 signaling. Nat Commun. (2017) 8:310. 10.1038/s41467-017-00411-z 28827520 PMC5566477

[37] Buchheit CL Weigel KJ Schafer ZT. Cancer cell survival during detachment from the ECM: multiple barriers to tumour progression. Nat Rev Cancer. (2014) 14:632–41. 10.1038/nrc3789 25098270

[38] Liu R Zhang T Zhu G Xing M. Regulation of mutant TERT by BRAF V600E/MAP kinase pathway through FOS/GABP in human cancer. Nat Commun. (2018) 9:579. 10.1038/s41467-018-03033-1 29422527 PMC5805723

[39] Xing M Liu R Liu X Murugan AK Zhu G Zeiger MA. BRAF V600E and TERT promoter mutations cooperatively identify the most aggressive papillary thyroid cancer with highest recurrence. J Clin Oncol. (2014) 32:2718–26. 10.1200/JCO.2014.55.5094 25024077 PMC4145183

[40] Liu X Qu S Liu R Sheng C Shi X Zhu G. TERT promoter mutations and their association with BRAF V600E mutation and aggressive clinicopathological characteristics of thyroid cancer. J Clin Endocrinol Metab. (2014) 99:E1130–6. 10.1210/jc.2013-4048 24617711 PMC4037723

[41] Liu X Bishop J Shan Y Pai S Liu D Murugan AK. Highly prevalent TERT promoter mutations in aggressive thyroid cancers. Endocr Relat Cancer. (2013) 20:603–10. 10.1530/ERC-13-0210 23766237 PMC3782569

[42] Hua K Jin J Zhang H Zhao B Wu C Xu H. MicroRNA-7 inhibits proliferation, migration and invasion of thyroid papillary cancer cells via targeting CKS2. Int J Oncol. (2016) 49:1531–40. 10.3892/ijo.2016.3660 27633373

[43] Yue K Wang X Wu Y Zhou X He Q Duan Y. microRNA-7 regulates cell growth, migration and invasion via direct targeting of PAK1 in thyroid cancer. Mol Med Rep. (2016) 14:2127–34. 10.3892/mmr.2016.5477 27430434

[44] Weinrich SL Pruzan R Ma L Ouellette M Tesmer VM Holt SE. Reconstitution of human telomerase with the template RNA component hTR and the catalytic protein subunit hTRT. Nat Genet. (1997) 17:498–502. 10.1038/ng1297-498 9398860

[45] Mocellin S Pooley KA Nitti D. Telomerase and the search for the end of cancer. Trends Mol Med. (2013) 19:125–33. 10.1016/j.molmed.2012.11.006 23253475

[46] Satyanarayana A Manns MP Rudolph KL. Telomeres, telomerase and cancer: an endless search to target the ends. Cell Cycle. (2004) 3:1138–50. 10.4161/cc.3.9.1152 15467446

[47] Li Q Li H Zhang L Zhang C Yan W Wang C. Identification of novel long non-coding RNA biomarkers for prognosis prediction of papillary thyroid cancer. Oncotarget. (2017) 8:46136–44. 10.18632/oncotarget.17556 28545026 PMC5542255

[48] Luo YH Liang L He RQ Wen DY Deng GF Yang H. RNA-sequencing investigation identifies an effective risk score generated by three novel lncRNAs for the survival of papillary thyroid cancer patients. Oncotarget. (2017) 8:74139–58. 10.18632/oncotarget.18274 29088774 PMC5650329

